# Supplementary material for: Fabrication of High-Acyl Gellan-Gum-Stabilized β-Carotene Emulsion: Physicochemical Properties and In Vitro Digestion Simulation
Source: Foods. 2022 Jun 14;11(12):1742. doi: 10.3390/foods11121742 (PMC9222914; doi:10.3390/foods11121742)
Supplement: Supplementary file 1 [file foods-11-01742-s001.zip › foods-1735016-supplementary.pdf]

Supplementary material

Fabrication of high acyl gellan gum stabilized *β*-carotene emulsion: physicochemical properties and in vitro digestion model

Table S1. Conditions of in vitro simulated digestion

**Process of simulated digestion**	**Conditions**	**Composition**	**Concentration**
simulated oral	T=37 °C	KCl	0.896g/L
digestion phase	pH=7.0	NaH_2PO_4	0.888g/L
(SODP)	Simulated time=3min	Na_2SO_4	0.57g/L
		NaCl	0.298g/L
		α-amylase	0.6g/L
simulated gastric	T=37 °C	NaCl	2g/L
digestion phase	pH=2.0	Pepsase	3.2mg/mL
(SGDP)	Simulated time =1h		
simulated intestinal	T=37 °C	Pancreatic enzyme	2.2mg/mL
digestion phase	pH=7.0	Bile salt of pig	44mg/mL
(SIDP)	Simulated time =2h	Saccharifying enzyme	140U
		$CaCl_2$	0.2442mg/mL
